# Virulence and Non-Virulence

**Published:** 1898-07-02

**Authors:** 


### VIRULENCE AND NON-VIRULENCE.

In the very interesting Croonian lectures now being delivered by Professor Sidney Martin before the Royal College of Physicians on the chemical products of pathogenic bacteria, an important point arises in regard to the difference in the products of the typhoid organism in its different conditions as to virulence. This matter of varying virulence is indeed one of the most important matters in that branch of pathology which deals with the microbic origin of infective diseases, for on the influences which determine virulence or non-virulence no doubt depends much which is at present dark as to the etiology of these diseases, their occasional latency, and their spread in an epidemic form. Professor Martin says that none of the ordinary cultures of the typhoid bacillus obtainable in laboratories will kill an animal. He obtained cultures from various laboratories in England, and found them all incapable of causing death. He then describes the methods adopted for obtaining the virulent microbe, and goes on to say that the virulence even of the bacillus as obtained direct from the spleen after death or during the course of the disease varies somewhat. Another matter of interest is that after injection of the sterile poison derived from the spleen—*i.e.*, the chemical poison free from microbes—there was a very distinct period of incubation, during which no effect on the body temperature or the weight was to be observed, and that not till the fifth day after injection did symptoms arise—a course very different from what we are given to associate with the action of a poison.

